# Characterizing the *Retinoblastoma 1* locus: putative elements for *Rb1* regulation by *in silico* analysis

**DOI:** 10.3389/fgene.2014.00002

**Published:** 2014-01-28

**Authors:** Mohammadreza Hajjari, Atefeh Khoshnevisan, Bernardo Lemos

**Affiliations:** ^1^Department of Genetics, Shahid Chamran University of AhvazAhvaz, Iran; ^2^Department of Genetics, School of Biological Sciences, Tarbiat Modares UniversityTehran, Iran; ^3^Molecular and Integrative Physiological Sciences Program, Department of Environmental Health, Harvard School of Public HealthBoston, MA, USA

**Keywords:** retinoblastoma, epigenetics, CpG islands, *in silico* analysis

## Abstract

Limited understanding of the *Rb1* locus hinders genetic and epigenetic analyses of Retinoblastoma, a childhood cancer of the nervous systems. In this study, we used *in silico* tools to investigate and review putative genetic and epigenetic elements of the *Rb1* gene. We report transcription start sites, CpG islands, and regulatory moieties that are likely to influence transcriptional states of this gene. These might contribute genetic and epigenetic information modulating tissue-specific transcripts and expression levels of *Rb1*. The elements we identified include tandem repeats that reside within or next to CpG islands near *Rb1*'s transcriptional start site, and that are likely to be polymorphic among individuals. Our analyses highlight the complexity of this gene and suggest opportunities and limitations for future studies of retinoblastoma, genetic counseling, and the accurate identification of patients at greater risk of developing the malignancy.

## Introduction

The retinoblastoma gene (*Rb1*) is one of the most widely studied tumor suppressors (Vogelstein and Kinzler, 2004). Retinoblastoma (RB) is a prototype cancer driven in large part by lesions in *Rb1*, a well-defined genetic element and clinical target. Point mutations, deletions, and epigenetic alterations in *Rb1* are also associated with a number of other malignancies (De La Rosa-Velázquez et al., 2007). Recent advances in genomics and epigenomics have made it possible to study RB in novel ways, with approaches combining multiple complementary techniques revealing key genetic and epigenetic steps at the origin of this malignancy (Reis et al., 2012).

Cryptic genetic and epigenetic variation in *Rb1* might contribute variation in the progression and drug response of RB tumors. It is plausible that differential penetrance and variation in the age of onset, which have been observed in patients with hereditary and non-hereditary RB, are attributed to epigenetic regulation of *Rb1* (Kanber et al., 2009). Three CpG islands (CpG106, 42, and 85) potentially involved in regulation of *Rb1* expression have been identified and investigated in detail (Greger et al., 1989). However, uncovering the genetic and epigenetic complexity of the *Rb1* locus remains challenging. This is in part due to a lack of complete understanding of the cis-regulatory elements controlling the expression of the gene. Furthermore, evidence of imprinted expression of *Rb1* suggests that epigenetic mechanisms might play a central role in the regulation of *Rb1* (reviewed in Reis et al., 2012). We expect that comprehensive analyses of the genetic and epigenetic properties of the human *Rb1* gene might reveal new aspects underlying its regulation. In this study, we have characterized a number of features of *Rb1* and presented some potential mechanisms that might be involved in regulation of this gene. Combining the results of several approaches and databanks will promote a better biological understanding of *Rb1*, and contribute toward improved clinical management and counseling of RB patients.

## Materials and methods

We combined a set of methods to identify putative functional elements in the *Rb1* locus. Our inferences are based on publicly available databases and re-analyses of experimental data. Table 1 lists the softwares used in this study. We defined the Genomic Region under Analysis (GRA) as a sequence that spans from 2 kb upstream of annotated Transcription start site (TSS) of *Rb1* to the end of the gene. This was based on previous studies which defined human putative promoter regions as sequences that correspond to −2000 to +1000 bp relative to the TSS (Marino-Ramirez et al., 2004).

**Table 1:** **Databases and softwares used in this study**.

**Application**	**Program/database**	**Reference/address**
Finding mRNA isoforms	Ace view	www.ncbi.nlm.nih.gov/IEB/Research/Acembly/
	UCSC	http://genome.ucsc.edu/
Expression analysis	Ace view	www.ncbi.nlm.nih.gov/IEB/Research/Acembly/
	Affymetrix exon array GNF Gene Expression Atlas2	http://genome.ucsc.edu/
Promoter detection	Hidden Markov Model	UCSC (http://genome.ucsc.edu/)
	CoreBoost_HM Promoter Prediction	UCSC (http://genome.ucsc.edu/)
	Promoter scan	www-bimas.cit.nih.gov/molbio/proscan/
	Promoter2	www.cbs.dtu.dk/services/Promoter
Alternative transcription start sites	DBTSS	http://dbtss.hgc.jp/
	Eponine	UCSC (http://genome.ucsc.edu/)
	SwithGear	UCSC (http://genome.ucsc.edu/)
Detection of CpGIs	UCSC	http://genome.ucsc.edu/
	Bona fide CGIs	http://epigraph.mpi-inf.mpg.de/download/CpG_islands_revisited/
	CpGProD	http://pbil.univ-lyon1.fr/software/cpgprod.html
	CpGcluster	http://bioinfo2.ugr.es/CpGcluster/
	CpG-MI tool	http://bioinfo.hrbmu.edu.cn/cpgmi/
	Weizmann Evolutionary CpG Islands	UCSC (http://genome.ucsc.edu/)
Estimation of the CGI's methylation status	Bona fide CGIs	http://epigraph.mpi-inf.mpg.de/download/CpG_islands_revisited/
Finding repeated sequences	Estimation of repeat variability	http://hulsweb1.cgr.harvard.edu/SERV/
	Repeat masker	http://genome.ucsc.edu/
Inspecting histone marks	UCSC	http://genome.ucsc.edu/
DNase I hypersensitive sites	UCSC	http://genome.ucsc.edu/
Transcription factor binding sites	CisRed	www.cisred.org/
	PReMode	http://genomequebec.mcgill.ca/PReMod/
	ENCODE	UCSC (http://genome.ucsc.edu/)
Prediction of insulator sites	CTCFBSDB	http://insulatordb.uthsc.edu

## Results

### Expression of *Rb1* and mRNA isoforms

**Figure 1:**
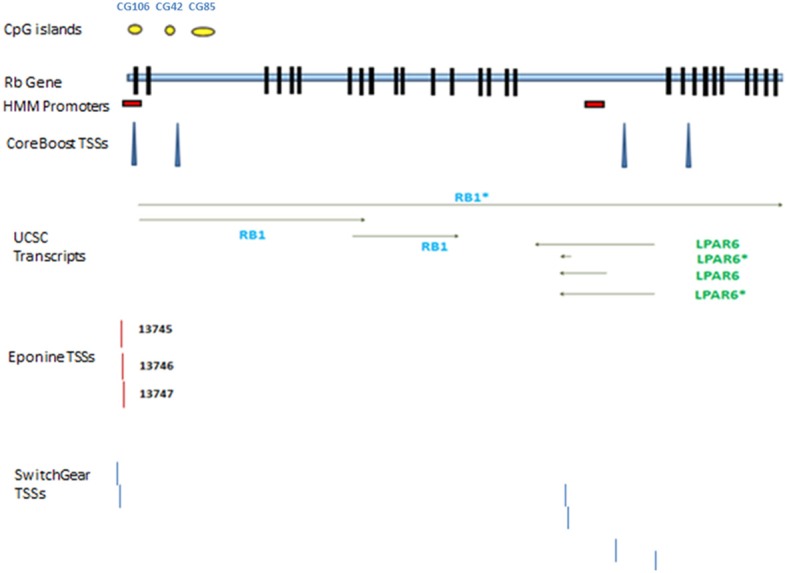
***Rb1* gene structure**. Transcribed RNAs from this locus identified by UCSC genome browser. The exons are represented by black blocks. Different promoters and transcription start sites of *Rb1* locus are shown. The diagram shows a schematic representation of results from different databases and programs which are described in the text. The yellow circles show CpG islands identified by UCSC genome browser. There are two red boxes which show the promoters identified by *HMM-Promoter* prediction algorithm. TSSs (Transcription start sites) are recognized by different algorithms such as *CoreBoost*, *Eponine*, and *SwitchGear*. *LPAR* is a gene within *Rb1*.

According to AceView, *Rb1* is expressed at 3.1 times the average gene. The database provides a comprehensive and non-redundant sequence representation of public mRNA sequences, and identified 33 potentially distinct GT-AG introns in *Rb1* (Thierry-Mieg and Thierry-Mieg, 2006). These result in 17 different mRNAs, 10 of which are produced through alternative splicing. There are 3 probable alternative promoters, 3 non-overlapping alternative last exons, and 3 validated alternative polyadenylation sites (http://www.ncbi.nlm.nih.gov/IEB/Research/Acembly/). One variant has a supporting clone (NM_000321.2) in Refseq database. According to the UCSC browser, there are three different transcripts, one of which is represented by Refseq (Figure 1). Finally, the *GNF Atlas* indicates that *Rb1* is expressed at variable levels across tissues (supplementary Figure 1).

### Promoters and TSSs

Chromatin state segmentation using Hidden Markov Model (HMM) (Pedersen et al., 1996) indicates that at least two promoters might be found in the *Rb1* region. One promoter is near the canonical TSS and another is within one of its introns. According to current annotation, there is a gene named *LPAR* (*P2RY5*) within this intron. Alternative splicing of *LPAR* results in multiple transcript variants. The second active promoter overlaps with TSS of *Rb1* (Figure 1). Promoter prediction with *CoreBoost_HM* identifies 4 hits in the GRA (Figure 1). *CoreBoost_HM* integrates DNA sequence features with epigenetic information to identify RNA polymerase II core-promoters (Wang et al., 2009). In addition, multiple TSSs were found using *Eponine* and *SwitchGear* (Figure 1). “*Eponine*” provides a probabilistic method for detecting TSS, with good specificity and positional accuracy (Down and Hubbard, 2002). “*SwithGear*” describes the location of TSSs throughout the genome along with a confidence measure for each TSS based on experimental evidence (http://genome.ucsc.edu/). Finally, the DBTSS database, which is based on the TSS sequencing method (TSS-Seq), suggests that distinct TSSs might be active in different cell lines (Table 2) (Yamashita et al., 2012). Altogether, the results point to alternative promoters and TSSs in the *Rb1* gene.

**Table 2:** **Transcription start sites (TSSs) identified in the DBTSS database for different cell lines**.

**Cell line**	**TSS position (positions are based on UCSC hg19)**
Hela	48878016
DLD1	48877884
Beas2B	48877877
Ramos	48877983
	48876242
MCF7	48877937

Table shows the cell lines (left column) and the position of TSSs in the Rb1 gene.

### Detection of CpGIs

**Figure 2:**
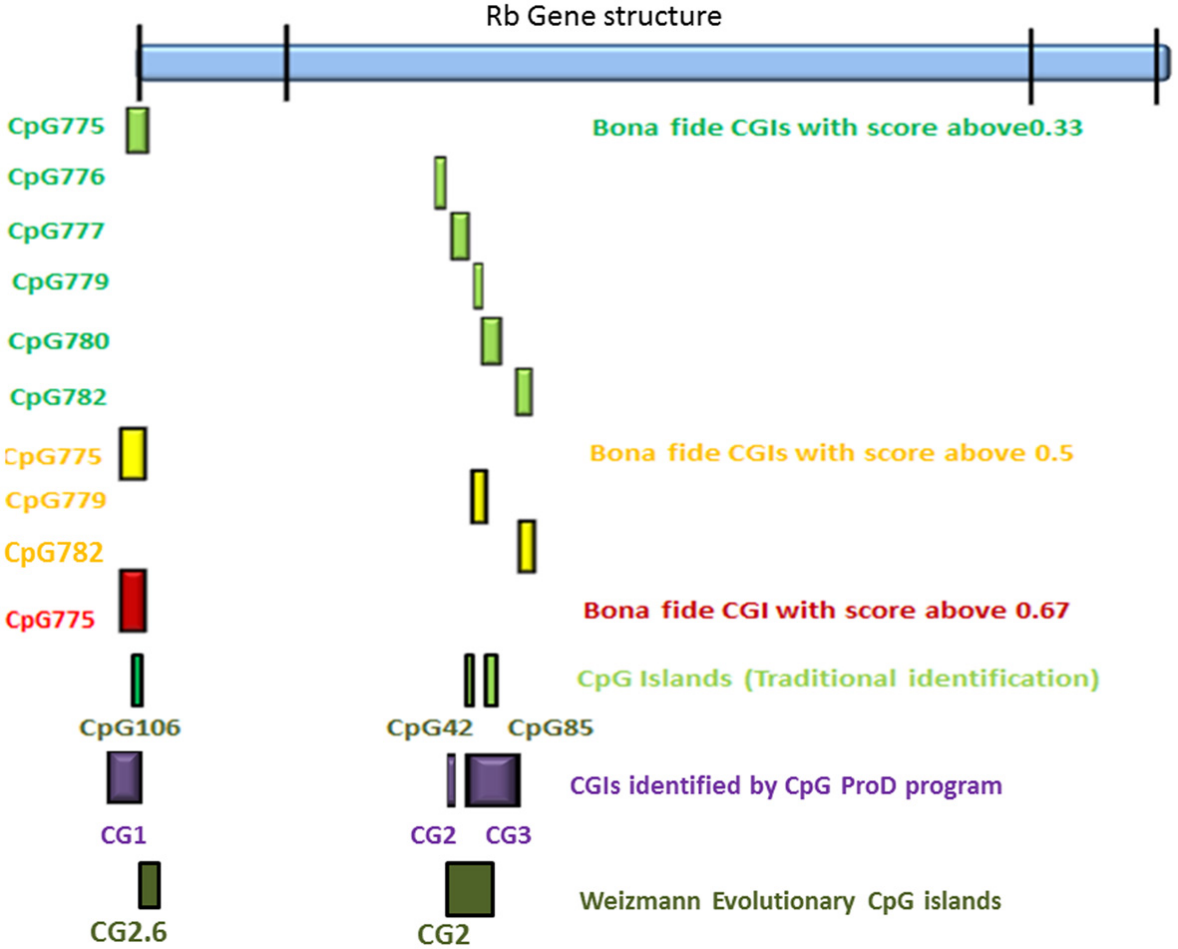
**The positions of CpG islands in the *Rb1* locus**. The first and last blocks in the schematic gene represent the first and fourth exons of *Rb1*, respectively. “Bona fide” strategy accounts for a number of functional CGIs and estimates their strengths (see scores in the figure). Also, *CpGProD* program predicts promoter- overlapping CGIs. “Weizmann CpG islands” predicts highly conserved CGIs. Although different methods were used, the results are largely concordant.

According to the UCSC browser searching criteria for CpGIs (traditional method), there were 3 CpG islands (Figure 2) in the *Rb1* (CGIs106, 42, and 85). UCSC identifies CpGIs of human genes using three criteria: (1) GC content greater than 50%, (2) length greater than 200 bp, and (3) large ratio between observed and expected number of CG dinucleotides (Gardiner-Garden and Frommer, 1987). Further analysis indicates additional putative segments containing CpGs. The “*bona fide*” strategy integrates genomic and epigenomic information to screen functional CGIs (Bock et al., 2007). We found eight *bona fide* CpGIs residing within the *Rb1* region (CGI 775-83). Three of them demonstrated positional overlap or neighborhood with three CpGIs predicted by traditional methods and previous studies. Only one of the CpGIs (106 in traditional finding and 775 in Bona fide CGIs) was near the canonical TSS of *Rb1*. The remaining CGIs were in intron 2 (Figure 2). Analysis of the targeted genomic region with the “*CpGProD*” program points to different CGIs over the length of *Rb1* (Figure 2 and Table 3). The program investigates prediction of promoter-overlapping CGIs with a longer length and greater CpGo/e ratio compared with non-overlapping start site CGIs (Ponger and Mouchiroud, 2002). Further, the “*CpG cluster*” program detects CpGIs based on the distance between neighboring CpGs. Because a minimum threshold length is not required, *CpG cluster* can find short but fully functional CGIs usually missed by other algorithms. In our study, most of the CpGs identified by this program overlap with the bona fide CGI regions (Table 3). Finally, the “Weizmann Evolutionary CpGIs” identified two different CpGIs (CpG2 and 2.6) (Figure 2). This custom track of UCSC predicts genome's regulatory elements with highly conserved sequences. Table 3 shows a comparison of the CpGIs positions identified by different programs.

**Table 3:** **Comparison of CpGs identified with different programs**.

**Regions (kb)**	**Traditional CpG finding**	**Bonafide CpGIs (B3 group)**	**CpGProD**	**CpGcluster**
1–3.5	CGI106: 1578-2619	CGI775:1429-2956	CG1:1370-3076	#1:1540-1710
				#2:1759-2619
				#3:2673-2898
10–10.5	No	No		#4:10015-10235
12–12.5	No	No		#5:12159-12302
14.5–16	CGI42:15076-15667	No	CG2:14857-15723	#6:15038-15446
				#7:15560-15667
				#8:15839-16103
16–17	CGI85:16754-17975	CGI779:16336-16550	CG3:16486-20182	#9:16592-16986
17–18		No		#10:17039-17211
				#11:17254-17430
				#12:17494-17738
				#13:17786-17975
18–19	No	No		#14:18458-18645
				#15:18807-19080
19–20.5	No	CGI782: 19195-19545		#16:19167-19443
				#17:19596-19672
				#18:19823-20089
155–165	No	No	CG4:163702-164409	#19:155929-156023
				#20:156294-156415
				#21:163774-164177

Although each algorithm has its own strategy, there are some concordances between the results. For simplicity, we have divided the Genomic Region under Analysis (GRA) into smaller segments (First column).

### Estimation of the CGI's methylation status

Several programs can be used to predict CGIs methylation status (Carson et al., 2008). The scores reflect the ability of each CGI to maintain its unmethylated state. All genomic CGIs are grouped into four sets: B1(0–0.33), B2(0.33–0.50), B3(0.50–0.67), and B4(0.67–1), whereby CGIs with combined scores >0.5 represent CGIs that are strongly associated with epigenetic regulatory function (http://epigraph.mpi-inf.mpg.de/download/CpG_islands_revisited/). Also, we evaluated two other indicators of methylation status in CGIs: the over-representation of CCGC motif within sequences of CpG islands (Bock et al., 2007) and the presence of H3K4me3 marks in CGIs (Su et al., 2010). We found three CpG islands (CpG775, 779, and 782) within groups B3 and B4. All these CpG islands had CCGC motif in their sequences. Also, we observed other regions which were methylated in different cell lines of ENCODE project (http://genome.ucsc.edu/cgi-bin/hgTracks?position=chr13:48875883-49056026&hgsid=347686961&wgEncodeHaibMethyl450=dense).

### Tandem repeats

By using “Estimation of Repeat Variability” toolkit, we found multiple tandem repeats in the GRA (Table 4). Three characteristics of the repeats (number of repeated units, unit length, and purity) were considered to produce a numeric “VARscore,” which correlates with repeat variability (Legendre et al., 2007). In our result, CGI-775, which includes the TSS of *Rb1* locus, is over a 3 bp unit VNTR. The sequence of this VNTR is: GCCGCCGCCACCGCCGCCGCTGCCGCCGCGGACCCCCGGCACCGCCGCCGCCGCC. Hence, longer alleles can add CpGs to the number of methylatable sites. Another tandem repeat identified by this software is downstream of CpGI number 6 recognized by CpG cluster. CpGI 6 was not found by bona fide as a functional island, but we observed that the CCGC motif is represented 4 times in the segment that includes CpGI 6 and the VNTR. Also, inspection for transcription factor binding sites in this segment by “TFSearch” software, indicates that there is CREB binding site motif in this region. Enrichment of representation of binding site of this transcription factor characterizes methylation free CpG islands (Tate and Bird, 1993; Sunahori et al., 2009).

**Table 4:** **Tandem repeats in *Rb1***.

**Consensus sequence**	**Start-end**
GCC^*^	2194–2246
CA^**^	14974–15038
TG	44625–44668
TG	104353–104389
AGTCATCTTCTACCAAACCTCACCTCCAGCATTGGGGAGCACACTTCAACACG	125368–126744
AAAC	128996–129033
TTCT	158141–158239

Repeats were recognized by the “Estimation of Repeat variability” toolkit and have Var score above 0.5. Positions are relative to the nucleotide in −2 kb of the canonical Rb1 transcription start site.

* Overlapped with CpG # 775.

** Neighborhood with CpG #6 identified by CpG cluster.

### Inspecting histone marks

We observed H3K4me1 and H3K4me3 through the annotated core *Rb1* promoter (supplementary Figure 2). The observation was made with data from the ENCODE project. H3K4me1 and H3K4me3 positive marks were mostly mirroring the acetylated histones. It is of note that the regions of histone marks mostly overlapped with CGI-775 and promoters identified by different programs.

### DNase I hypersensitive sites (DNase I HS)

We used *DNase Clusters* track in UCSC genome browser. In the *Rb1* promoter, positions of the DNase I HS sites vary depending on cell line assayed. Notably, DNase I HS sites are mostly mapped to CGI_775, which overlaps with CG106. Also, we found that some of these hypersensitive sites are overlapped with or adjacent to other predicted CpGIs.

### Transcription factor binding sites

“*CisRed*” and “*PReMode*” databases were used to detect the boundaries of regulatory regions and TFBs motifs distribution. CisRed summarizes conserved sequence motifs identified by genome scale motif discovery, similarity, clustering, co-occurrence, and coexpression calculations (Robertson et al., 2006). The algorithm used in PReMode predicts transcriptional regulatory modules (Ferretti et al., 2007) in which a number of transcription factors can bind and regulate expression of nearby genes (Ben-Tabou De-Leon and Davidson, 2007; Teif, 2010). There were three modules concentrated within or next to CpGs around TSS. Two modules were near the canonical TSS. Finally, the ENCODE results in UCSC point to regions with abundant binding of transcription factors.

### Insulator sites

A comprehensive collection of experimentally determined and computationally predicted CTCF binding sites have been curated in the “CTCFBSDB” database (Bao et al., 2008). We observed 6 putative sites for CTCF binding in GRA, two of which are located in CpGI-775 (Table 5).

**Table 5:** **CTCF binding motifs within the Genomic Region under Analysis**.

**Motif sequence**	**Motif start location**
CCGGCCTGGAGGGGGTGGTT	1796
GGAACTGCA	2597

The positions are relative to −2 kb of the canonical transcription start site of the Rb1 gene.

## Discussion

Neural progenitor cells dynamically interact with their environment (Jones and Laird, 1999). The expanded two hit hypothesis proposes that both genetic and epigenetic aberrations are involved in silencing of tumor suppressor genes in cancers such as RB (Jones and Laird, 1999). Studies have shown the role of epigenetic mechanisms in *Rb1* regulation (Reviewed in Reis et al., 2012), but the exact elements and their relation with *cis* regulatory elements already identified as important for *Rb1* expression has remained elusive. Here we used *in silico* analyses and databases to identify and summarize putative regulatory elements that might contribute to *Rb1* regulation. Identification of these elements suggests new venues for understanding *Rb1* expression and its contribution to disease states. The analyses reinforce the notion that a variety of distinct epigenetic and genetic elements are involved in the control of the activity of the human *Rb1* gene.

A study by Greger et al. (1989) was among the first to provide evidence that changes in the methylation of *Rb1* might play a role in the emergence and progression of RB tumors. They found that CpG106, which overlaps the *Rb1* promoter and exon E1, is methylated in some RB cases. Two other CpGs (CpG 42 and 85) were investigated in other studies. Kanber et al. (2009) observed that an alternative transcript of *Rb1* is preferentially expressed from the maternal allele. It seems that imprinted expression of *Rb1* is linked to a differentially methylated CpG island in intron 2 of this gene (CpG-85) (Kanber et al., 2009). Also, it has been reported that CpG 42 is biallelically methylated, whereas CpG-106 is biallelically unmethylated (Buiting et al., 2010).

We identified additional CpG islands in the *Rb1* locus and sought to assess their epigenetic state by evaluating other data such as co-occurrence of histone modifications, DNAse 1 sensitivity, transcription factor binding sites, and presence of genomic insulators. One possibility is that these genetic and epigenetic features cooperate to fine tune *Rb1* regulation. Our observations highlight two points. First, the *Rb1* locus includes multiple genomic elements exhibiting potential sensitivity to differential DNA methylation and histone modification. Independent tools identified multiple CpG islands in the locus. In spite of differences between softwares, all of them pointed to multiple CpGs, some of which were corroborated by multiple lines of evidence. These are promising targets for downstream functional analysis. Second, repeats occur within or next to some CpG islands. Hence we expect that the methylation status of the *Rb1* regulatory regions in genomes of different individuals might be affected by repeat number variations in nearby sequences. The potential contribution of these regions to the epigenetic regulation of *Rb1* alleles might be worthy of further study. Individual methylation profile might lead to variable expressivity and penetrance in different patients.

Several mammalian genes contain more than a single TSS (Valen et al., 2009) and *Rb1* does not appear to be an exception. Genes with alternative promoters, often display only one promoter with a CGI (Cheong et al., 2006). On the other hand, most of the putative alternative promoters of *Rb1* are distributed in or next to putative CpG islands. Since methylation sensitive regions carry distinctly different information about gene expression and exhibit different sensitivity to regulatory signals, this type of positioning should not be neglected. Besides, DNA methylation appears to play a significant role in differential usage of alternative promoters and be related to functional diversification between CpGI-containing promoters and CpGI-less promoters. Furthermore, chromatin marks and transcription elements such as enhancers or insulators could cause differential expression levels in *Rb1* or even differential usage of the gene's TSSs. The presence of multiple regulatory elements within the locus confers combinatorial control of regulation through which the number of unique expression states can increase (Maston et al., 2006).

The distribution and amount of histone marks like H3K4me1-3 provide a basis for nucleosome positioning in the *Rb1* locus. H3K4me1 is associated with enhancers and DNA regions downstream of TSSs. The H3K4me3 histone mark is associated with promoters that are active or poised to be activated (Karliæ et al., 2010). This histone mark seems to be an indicator of functional CpG islands (Su et al., 2010). We observed an overlap between the regions including this mark and predicted CpGIs (supplementary Figure 2).

It has been reported that DNA methylation correlates with DNase 1 hypersensitivity (Crawford et al., 2006). We found that DNase 1 hypersensitive regions mapped to CGI_775. This CpG island overlaps with the canonical promoter of *Rb1* and this observation is in agreement with studies indicating that regulatory regions in the promoters tend to be DNase sensitive (Crawford et al., 2006). Noteworthy, we observed several CTCF binding sites in the *Rb1* locus. In vertebrates, the transcription regulator CCCTC-binding factor (CTCF) is the only *trans*-acting factor that is a primary part of insulator sequences that block the interaction between enhancers and promoters (Ohlsson et al., 2001). Hence, CTCF is at the core of the machinery that exerts epigenetic control of diverse imprinted loci and participates in promoter activation and repression. Evidence points toward a role for the 11-zinc finger CCCTC-binding factor (CTCF) in the establishment of DNA methylation free zones and the regulation of cell cycle–related genes (Tang et al., 2002; Filippova et al., 2005). CTCF-bound insulators separate transcriptionally active and silent chromatin domains, with their function depending strongly on the local status of DNA methylation and chromatin modifications. It has been suggested that active genes have a DNA fragment with insulator properties and CTCF binding sites in their 5' ends (Filippova et al., 2005).

Numerous experimental and clinical studies investigate the role of DNA methylation and other epigenetic marks in human diseases (Kanwal and Gupta, 2012). However, in spite of genome-wide patterns, the association between genomic polymorphisms and altered epigenetic status of specific genes is elusive. One interesting possibility is that genetic variations in the *Rb1* gene (including VNTRs) might contribute to the methylation status of the region. Hence, experimental methylation analysis would benefit most if coupled with the sequencing of primary genomic samples. Furthermore, genetic variations in repetitive segments not usually targeted in mutation screens might enable a better understanding of unexpected confounders due to personal genome variation. The proposed set of *Rb1* regulatory elements offers venues to understand the developmental dynamics and individual variation in the expression of the *Rb1* gene. Altogether, we expect that interactions between genetic and epigenetic elements of *Rb1* might cause tissue-specific alternative transcripts, different expression level, and possibly variable penetrance and disease severity in patients with RB.

### Conflict of interest statement

The authors declare that the research was conducted in the absence of any commercial or financial relationships that could be construed as a potential conflict of interest.
