# Supplementary material for: Characterizing the Retinoblastoma 1 locus: putative elements for Rb1 regulation by in silico analysis
Source: Front Genet. 2014 Jan 28;5:2. doi: 10.3389/fgene.2014.00002 (PMC3904107; doi:10.3389/fgene.2014.00002)
Supplement: Supplementary file 1 [file DataSheet1.PDF]

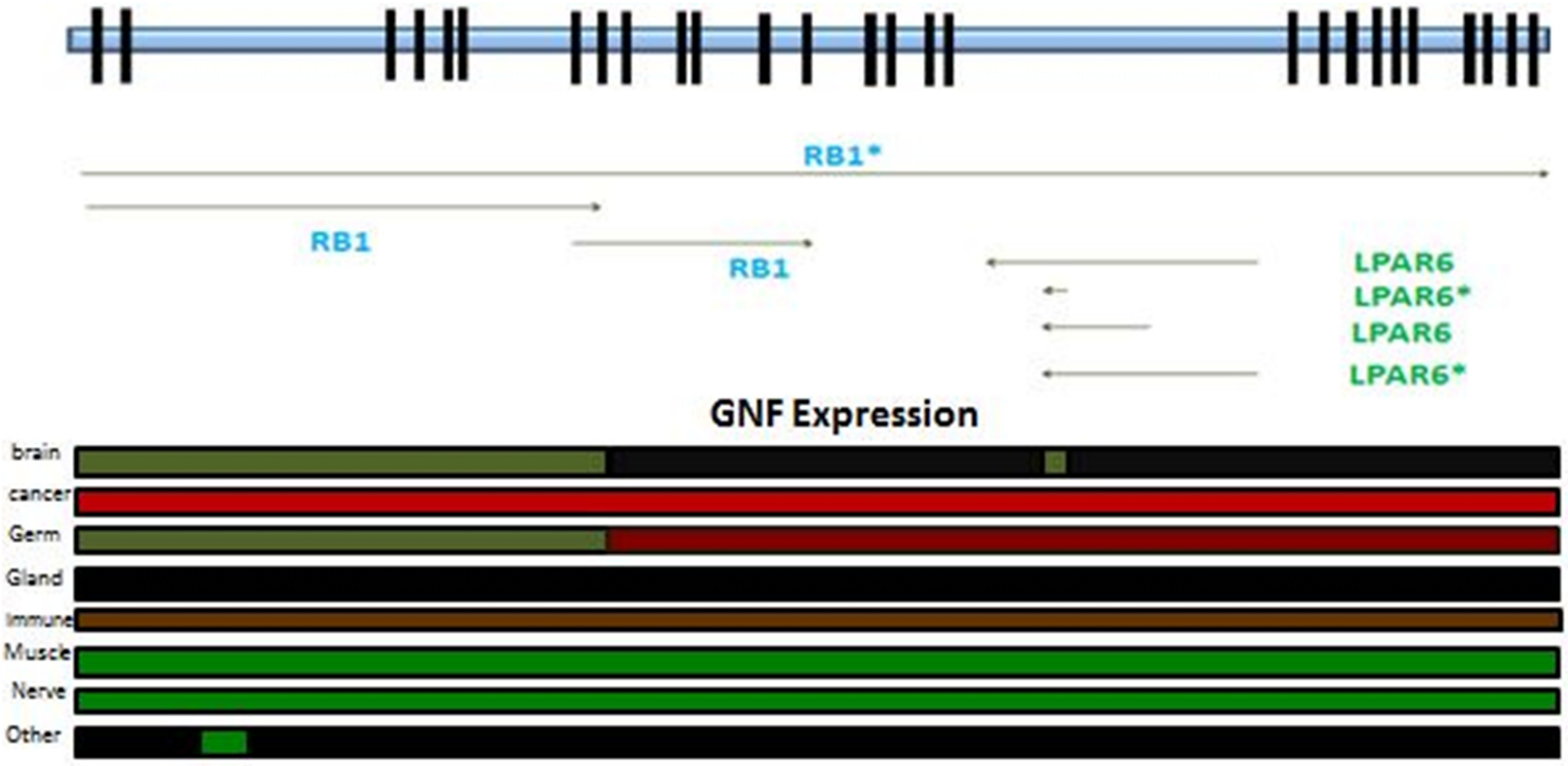

Sup. Fig. 2) Schematic view of the results drawn from GNF Gene Expression Atlas 2 for Rb1 in different human tissues. Red indicates overexpression in the tissue and green indicates under-expression.

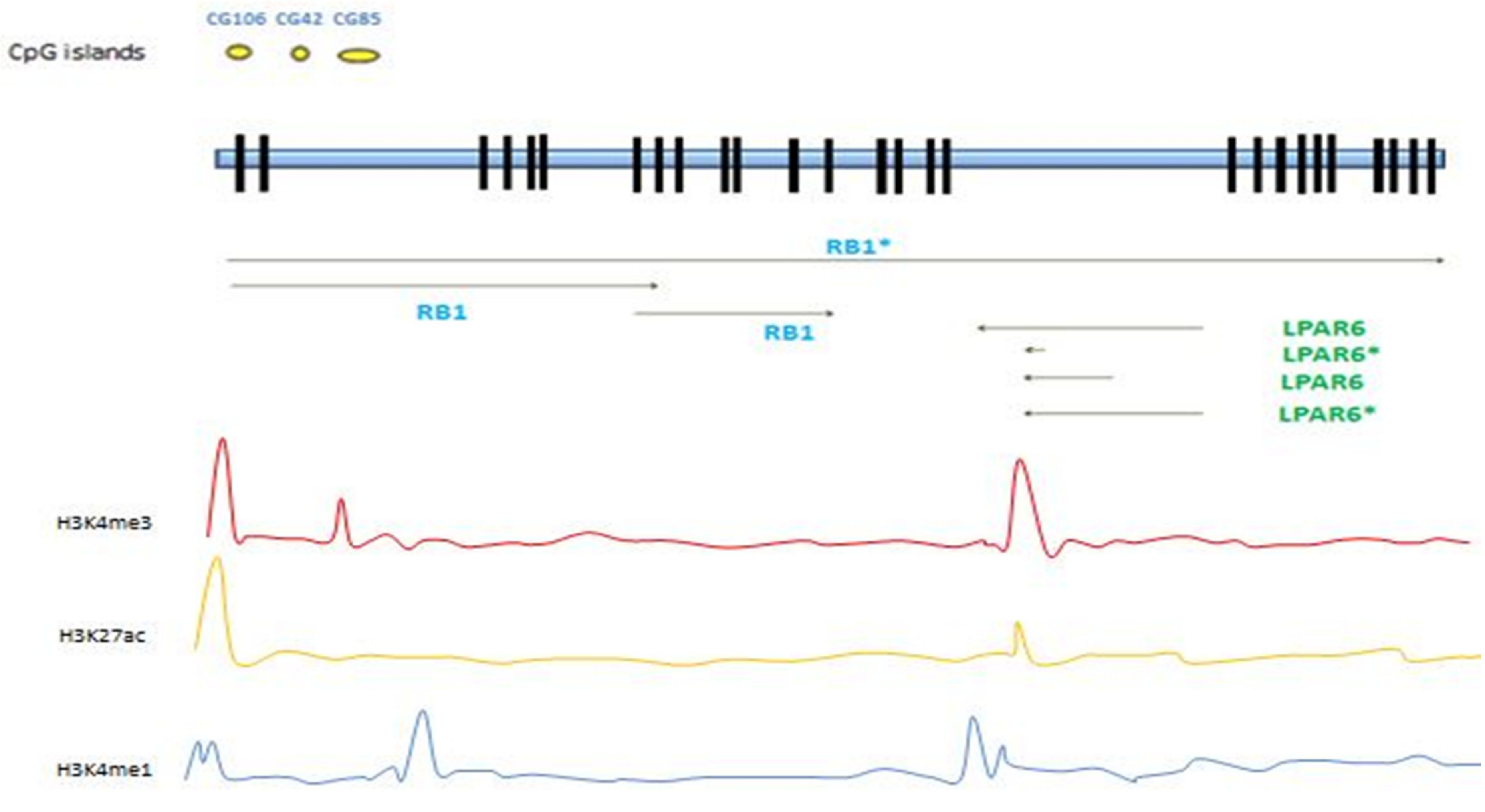

Sup. Fig. 2) Schematic view of the results drawn from ENCODE Consortium combinatorial data of H3K4me1 and H3K4me3 for different cell lines. These histones are marks of active promoters. These marks are compared with H3K27ac mark for the interested region.
